# Fibrous Variant of Hashimoto's Thyroiditis as a Diagnostic Pitfall in Thyroid Pathology

**DOI:** 10.1155/2013/308908

**Published:** 2013-12-05

**Authors:** G. Iannaci, R. Luise, P. Sapere, V. Coluccino, A. Ronchi, A. Faggiano, V. Marotta, A. Colao, S. Spiezia

**Affiliations:** ^1^Department of Pathology, S. Maria del Popolo degli Incurabili Hospital ASL NA1, 80138 Naples, Italy; ^2^Department of Clinical Medicine and Surgery, University Federico II of Naples, 80131 Naples, Italy; ^3^Department of Surgery, Ultrasound Guided & Neck Pathologies Surgery Unit, S. Maria del Popolo degli Incurabili Hospital ASL NA1, 80138 Naples, Italy

## Abstract

Fibrous variant of Hashimoto's thyroiditis is a rare condition occurring in about 10% cases, mainly middle age people. It is characterized by an extensive fibrous proliferation without extension into the surrounding structures. A 55-year-old female was referred to our department for an unexplained onset of cervical discomfort. She presented a voluminous goiter of hard consistence, dyspnea and dysphagia. Given the compressive symptoms and the non-diagnostic result of the biopsy, a total thyroidectomy was performed. Microscopically the thyroid parenchyma was characterized by broad bands of fibrosis with severe atrophy of thyroid follicles and lymphocytic inflammatory infiltrate distributed within and around the lobules. In view of the morphological and immunohistochemical findings, a diagnosis of HTFV was made. The fibrosclerotic process is the key feature of several thyroid diseases so that the clinician and the pathologist have to consider that many diagnostic pitfalls can occur in this field. The differential diagnosis between HTFV and RD is sometimes arduous due to the partial clinical and morphological overlapping and to the poor efficacy of conventional cytology as well as pre-surgical biopsy. Considering these features, histological examination is mostly mandatory.

## 1. Introduction 

Hashimoto's thyroiditis (HT) is the most common inflammatory condition of the thyroid gland. It is an autoimmune disorder manifesting predominantly in middle-aged women. Clinically, it causes a painless, diffused thyroid inflammation, associated with the presence of elevated titers of antithyroid antibodies and hypothyroidism in the majority of cases. In addition to the classic variant of HT, several other subtypes have been identified, such as the fibrous variant. Fibrous variant of Hashimoto's thyroiditis (HTFV) is a rare condition occurring in about 10% cases, mainly middle age people. It is characterized by an extensive fibrous proliferation without extension into the surrounding structures [1–3].

Riedel disease (RD) is not a real thyroiditis but rather a form of fibroinflammatory process of the thyroid involving also the soft periglandular tissues. Its incidence, estimated from large series of thyroidectomies, varies between 0.04% and 0.3% [3]. Clinically, it is characterized by a stony hard, enlarged thyroid with compression symptoms due to tracheal and esophageal involvement. Hypothyroidism and autoantibodies positivity are frequent findings in RD. Because of clinical and histological overlapping between these two pathological conditions, the results of diagnostic imaging, laboratory tests, and cytology studies are often similar [1–3]. 

The relationship between HTFV and RD remains unclear. Basing on morphologic features, they seem to be different variants of the same disease but are commonly considered as two distinct clinic-pathological entities. To date, it is still not clear if RD is a primary autoimmune process or a primary fibrotic disease. The frequent presence of thyroid autoantibodies (observed in 67% of RD) and lymphoid infiltration might indicate an autoimmune disease [4]. Nevertheless, it has been recently assumed that RD is part of a spectrum of IgG4 correlated syndromes and it might represent just one manifestation of a systemic fibrosclerotic process [5].

Here the authors reported a case of HTFV in view of the rarity of this diagnosis as well as the difficulties in making a differential diagnosis with RD. It is emphasized that morphological findings and immunological features are central points in the achievement of the diagnosis of HTFV.

## 2. Case Report 

A 55 -year-old female was referred to our department for an unexplained onset of cervical discomfort. She presented a voluminous goiter of hard consistence, dyspnea, and dysphagia. The laboratory tests showed normal levels of thyroid hormones FT3 and FT4 (euthyroid patient) and elevated antibodies against Tg and TPO. VES was elevated. Calcitonin serum levels were in the normal range. Ultrasound examination using a 10 MHz linear probe (Esaote, Genoa, Italy) revealed an increased thickness of prethyroid muscles, first of all the sternothyroid and sternohyoid muscles, with regular echogenicity of the muscular pattern. Thyroid gland appeared enlarged (right antero-posterior diameter: 28 mm, left anteroposterior diameter: 29 mm) and the echogenicity considerably reduced (echogenicity comparable to that of prethyroid muscles). The boundaries of the gland appeared linear and not well dissociable from the superficial muscular plans.

Thyroid parenchyma was inhomogeneous due to the presence of hypoechoic areas surrounded by hyperechoic rib (due to ineffective previous laser ablative treatments). There was no evidence of well-defined nodules and the intraglandular vascularity was scanty Figures 2(a) and 2(b). There were signs of compression of the tracheal vector; common carotid was surrounded by the glandular mass and internal jugular vein was laterally pushed.

CT and MRI scan confirmed the enlargement of the thyroid, especially of the left lobe, that extended down occupying part of the superior mediastinum with contralateral dislocation and modest compression of the trachea. The parenchymal structure was inhomogeneous with an evident hypodense area situated in the left lobe. The regional lymph nodes showed increased size and normal density.

**Figure 1:**
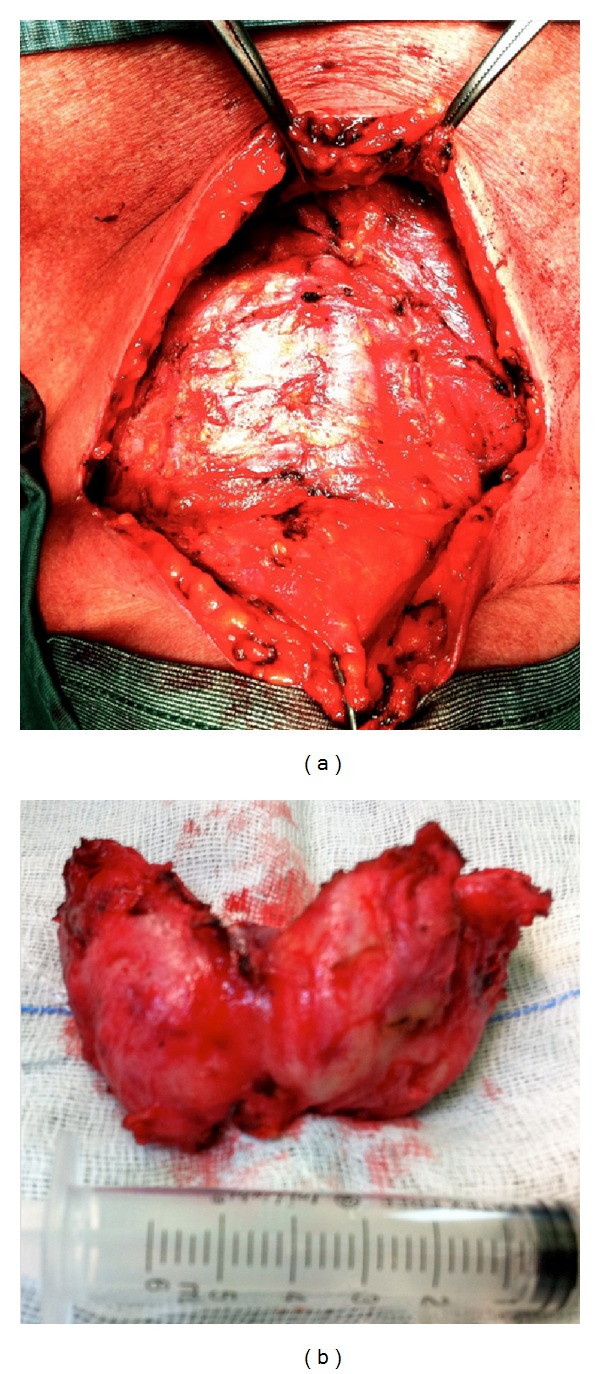
A macroscopic photograph. (a) Intraoperative image of the prethyroid muscular layer. (b) Thyroidectomy specimen.

**Figure 2:**
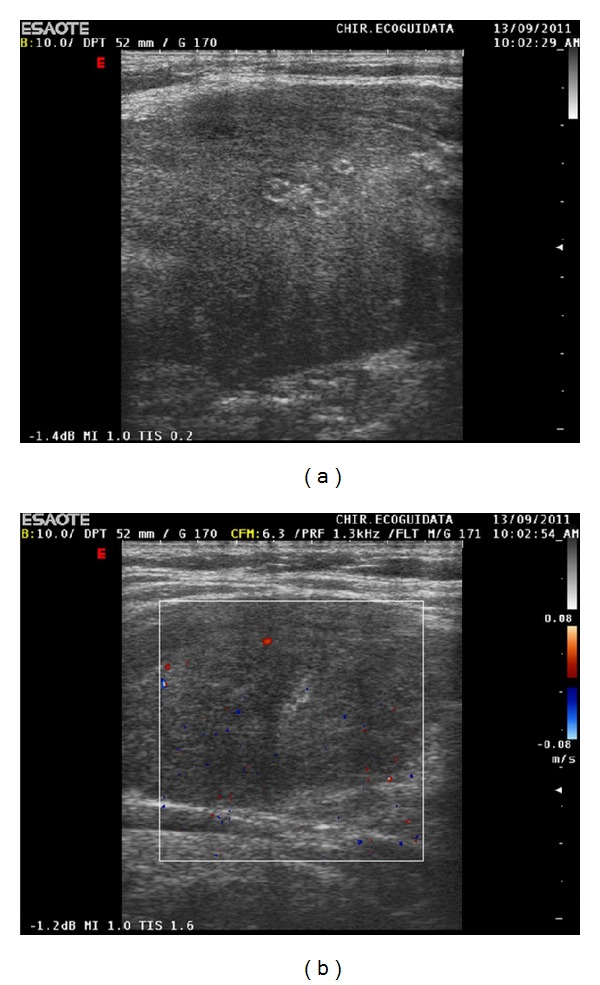
Ultrasound examination. (a) Longitudinal scan of the thyroid right lobe; the gland echogenicity is similar to that of the strap muscle. The postablative laser scares are clearly visible as “target like” focal areas inside the gland. (b) The color Doppler examination reveals a poor or absent vascular pattern.

**Figure 3:**
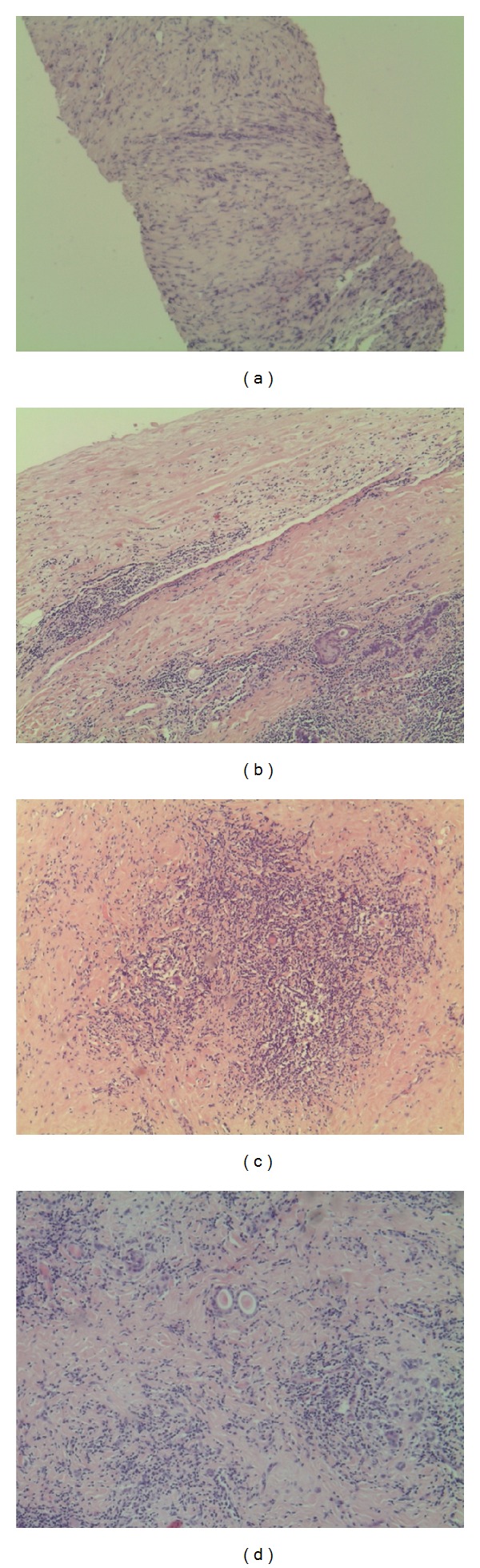
(a) H&E staining (4x): fine needle thyroid biopsy contained an admixture of diffuse chronic lymphocytic infiltration and dense fibrous tissue with a total absence of thyroid follicles. (b) H&E staining (20x): thyroid gland with capsule easily dissociable from the periglandular tissues and follicular cells in squamous metaplasia; (c),(d) H&E staining (20x): thyroid tissue with residual follicles immersed in dense fibrotic stroma and surrounded by diffuse chronic lymphocytic inflammation.

These clinical and instrumental features, especially the dislocation of the trachea and the apparent indissociability from the contiguous structures, were consistent with a diagnosis of RD rather than HTFV. Since cytology is rarely diagnostic in this kind of lesion, a fine needle thyroid biopsy was made to obtain a definitive diagnosis before the surgery. The histological picture contained an admixture of diffuse chronic lymphocytic infiltration and dense fibrous tissue with a total absence of thyroid follicles Figure 3(a). Given the compressive symptoms and the nondiagnostic result of the biopsy, which did not allow to exclude malignancy, a total thyroidectomy was performed one month later. Thyroid gland was enlarged with increased consistency and prethyroid muscles increased in thickness, being hard and poorly dissociable from the thyroid capsule Figures 1(a) and 1(b). Nevertheless, the gland was easily dissociable from the periglandular tissues, with a clear identification of the recurrent nerves and the parathyroid glands so that the surgeon was able to spare them.

Grossly, the patient presented a small fibrotic gland: right thyroid lobe was 5.0 × 3.0 × 1.2 cm, left lobe was 6.5 × 3.2 × 1.5 cm, and isthmus was 1.5 × 1.0 cm. On cut sections, it was firm but was not so hard as carcinoma, grayish white in color and fibrotic. It was extensively sampled. The tissue samples were fixed in formalin, then routinely processed, and embedded in paraffin. Microscopically, the entire gland showed an architectural destruction with the total loss of the normal lobular pattern. Thyroid parenchyma was characterized by broad bands of fibrosis with severe atrophy of thyroid follicles and lymphocytic inflammatory infiltrate distributed within and around the lobules. Of relevance, there were granulomatous microabscesses with central necrosis, which represent crucial features able to distinguish between RD and HTFV. Multiple areas of necrosis, cystic areas, and prominent squamous metaplasia of the follicular cells were seen, as a sign of longstanding HT. The massive fibrosis did not extend beyond the thyroid capsule to the adjacent muscle and surrounding tissues, further excluding the diagnosis of RD Figures 3(b), 3(c), and 3(d). The immunohistochemistry showed a widespread lymphocyte population made up of B cells (positive for CD20) with abundance of plasma cells (positive for CD138) and T lymphocytes with an increased CD8/CD4 ratio. These results allowed to exclude the diagnosis of thyroid lymphoma. Moreover, the negative results of galectin 3, CK19, and Calcitonin at immunohistochemistry excluded the diagnosis of malignancy, supporting the hypothesis of a benign lesion. In view of these morphological and immunohistochemical findings, a diagnosis of HTFV was made.

The patient was routinely monitored and she did not manifest dysphonia or alteration of blood calcium after the surgery. She is actually treated with L-thyroxine 150 mcg/day and she has a good quality of life.

## 3. Discussion 

Conventional cytology has a limited role for the differential diagnosis of HTFV. Indeed, in most cases, acellular or paucicellular, non-diagnostic smears are obtained [2, 3, 6, 7]. The fibrosclerotic process is the key feature of several thyroid diseases so that the clinician and the pathologist have to consider that many diagnostic pitfalls can occur in this field. Considering the similar clinical and morphological features, histological examination is mostly mandatory for distinguishing HTFV from other similar thyroid diseases. Therefore, a close cooperation between the clinician and the pathologist is necessary to avoid diagnostic errors [8–11]. The main differential diagnosis includes malignant tumors like the diffuse sclerosing variant of papillary carcinoma, the medullary carcinoma, paucicellular variant of anaplastic carcinoma and sarcomas, and large cell malignant lymphoma, Hodgkin disease nodular sclerosis type. These lesions may present large areas of sclerosis in the thyroid parenchyma. The benign diseases that have to be excluded for the differential diagnosis with HTFV are, paraganglioma-like adenoma, solitary fibrous tumor, or long term idiopathic myxedemas, which are characterized by fibrosis and atrophy with total loss of glandular pattern [3]. Sometimes sclerotic modifications of the thyroid gland with nuclear atypias of follicular cells, enlarged perithyroid blood vessels with thick fibrotic walls, might be seen, months or years after the exposition to radiation therapy [12].

Differential diagnosis between HTFV and RT is based on histological criteria established by Beahrs et al. then validated by Meijer and Hausman and Schwaegerle et al. [13–15].

In HTFV the fibroinflammatory process involves a part or the whole gland and it does not include the adjacent tissues. There is an inflammatory population, made up mainly of lymphocytes, with occasional formation of germinal centers, mixed with oncocytes and sometimes with granulomas with a variable number of giant cells. Of note, granulomas are absent in HTFV [16].

The differential diagnosis between HTFV and RD should be performed before surgery as the treatment is different. In RD, many authors suggest that surgical therapy should be preceded by steroid therapy in order to reduce the fibrosclerosis thus allowing a more effective surgery and achieving a complete remission. Otherwise, in HTFV the medical treatment includes just the somministration of L-thyroxine in consequence of total thyroidectomy.

In conclusion, this case report shows that the differential diagnosis between HTFV and RD is sometimes arduous due to the partial clinical and morphological overlapping and the poor efficacy of conventional cytology as well as presurgical biopsy. In the patient described here, the differential diagnosis was achieved only by an accurate histological examination.

## Abbreviations

HT: Hashimoto's thyroiditis
HTFV: Fibrous variant of Hashimoto's thyroiditis
RD: Riedel disease
CT: Computed tomography
MRI: Magnetic resonance imaging.
